# Biodegradation of Mixed PAHs by PAH-Degrading Endophytic Bacteria

**DOI:** 10.3390/ijerph13080805

**Published:** 2016-08-09

**Authors:** Xuezhu Zhu, Xue Ni, Michael Gatheru Waigi, Juan Liu, Kai Sun, Yanzheng Gao

**Affiliations:** Institute of Organic Contaminant Control and Soil Remediation, College of Resource and Environmental Sciences, Nanjing Agricultural University, Nanjing 210095, China; zhuxuezhu@njau.edu.cn (X.Z.); nixue@bgechina.cn (X.N.); mgatheru94@gmail.com (M.G.W.); liujuan@njau.edu.cn (J.L.); 2013203015@njau.edu.cn (K.S.)

**Keywords:** endophytic bacteria, *Pseudomonas* sp., *Stenotrophomonas* sp., 16S rRNA gene, biodegradation

## Abstract

Endophytic bacteria can promote plant growth, induce plant defence mechanisms, and increase plant resistance to organic contaminants. The aims of the present study were to isolate highly PAH-degrading endophytic bacteria from plants growing at PAH-contaminated sites and to evaluate the capabilities of these bacteria to degrade polycyclic aromatic hydrocarbons (PAHs) in vitro, which will be beneficial for re-colonizing target plants and reducing plant PAH residues through the inoculation of plants with endophytic bacteria. Two endophytic bacterial strains P_1_ (*Stenotrophomonas* sp.) and P_3_ (*Pseudomonas* sp.), which degraded more than 90% of phenanthrene (PHE) within 7 days, were isolated from *Conyza canadensis* and *Trifolium pretense* L., respectively. Both strains could use naphthalene (NAP), PHE, fluorene (FLR), pyrene (PYR), and benzo(*a*)pyrene (B(*a*)P) as the sole sources of carbon and energy. Moreover, these bacteria reduced the contamination of mixed PAHs at high levels after inoculation for 7 days; strain P_1_ degraded 98.0% NAP, 83.1% FLR, 87.8% PHE, 14.4% PYR, and 1.6% B(*a*)P, and strain P_3_ degraded 95.3% NAP, 87.9% FLR, 90.4% PHE, 6.9% PYR, and negligible B(*a*)P. Notably, the biodegradation of PAHs could be promoted through additional carbon and nitrogen nutrients; therein, beef extract was suggested as the optimal co-substrate for the degradation of PAHs by these two strains (99.1% PHE was degraded within 7 days). Compared with strain P_1_, strain P_3_ has more potential for the use in the removal of PAHs from plant tissues. These results provide a novel perspective in the reduction of plant PAH residues in PAH-contaminated sites through inoculating plants with highly PAH-degrading endophytic bacteria.

## 1. Introduction

Greatly increasing accumulations of polycyclic aromatic hydrocarbons (PAHs) have been found in multiple environments, including arable soils [1], urban lands [2,3], forests, and grasslands [4]. As noted in previous reports, the PAH levels were 80–7264 μg/kg in Poland arable soils [5], 1.08 and 6.25 mg/kg in two contaminated arable lands in Tianjing, China [1], 83.3–7220 μg/kg in the main urban areas of Shanghai, China [3], and 127–10,600 μg/kg at an electronic waste dismantling site in Guangzhou, China [6]. Because PAHs accumulate in food chains, reflecting the high hydrophobicity and affinity of these molecules for fatty tissues, the PAH contents in plants are increased. PAH levels ranging from 92 to 1454 μg/kg dry weight were detected in contaminated vegetables in Italy [4], and the PAH levels in some vegetables grown near electronic waste recycling sites in South China ranged from 199 to 2420 μg/kg [6], suggesting that the contamination of PAHs is a large threat to the safety of human health and the survival of wild animals.

Several studies have been conducted for the removal of PAHs from contamination sites. Microbial degradation has been touted as one of the main applications for PAH remediation in the environment [7]. Different consortia with different PAH-degrading capacities were enriched from tsunami-inundated sediments and composed of diverse bacteria [8]. Several bacteria with high capacities for degrading PAHs have been isolated [9]. A bacterial consortium from an oil contaminated site could degrade PAHs faster than alkanes and it was identified that *Burkholderia* played a key role in this rapid degradation of aromatic compounds [10]. Among these strains, a *Streptomyces* sp. isolated from oil-contaminated soil in India by Balachandran et al. [11] could remove naphthalene (NAP); *Rhodococcus* sp. P14, isolated by Song et al. [12] could remove phenanthrene (PHE), pyrene (PYR), and benzo(*a*)pyrene (B(*a*)P). A *Sphingobium* sp. strain FB3, isolated by Fu et al. [13], could degrade PHE, anthracene (ANT), fluoranthene (FLR), PYR, and B(*a*)P in a mixture of PAHs. However, some challenges remain for the application of highly PAH-degrading bacteria for the removal of PAHs from plants grown in PAH-contaminated sites.

Synergistic interactions between plants and microbial communities in the rhizosphere and interior plant tissues have been demonstrated to be effective for recalcitrant organic compounds [14]. Bacosa et al. reported that different consortia from mangrove sediments which could possibly associated with mangrove roots were isolated and mainly composed of *Pseudomonas* and *Burkholderia* [15]*.*

Most bacteria could not effectively colonize plant tissues and degrade PAHs in planta, reflecting the failure of these organisms to compete effectively with native plant microorganisms. Remarkably, plant-endophytic bacteria symbioses generate nutrients, and the niche provided by plants for bacteria, protects these organisms from competition with other native bacteria [16]. Endophytic bacteria can promote plant growth, induce plant defence, and increase plant resistance to organic contaminants [7,17,18]. Previous studies have reported that persistent organic pollutant (POPs)-degrading endophytic bacteria have capacities for enhancing the bioremediation of environments contaminated with POPs [19]. Moreover, endophytic bacteria have many positive effects on plant establishment and survival in heavily POP-contaminated soils, including increasing nutrient uptake [20], improving plant tolerance of POPs [16], and degrading POPs in plant tissues [21], affecting the activities of plant enzymes and secreting hormones, siderophores, and other organic compounds [22].

More PAH-degrading endophytic bacterial strains need to be isolated to reduce PAH contamination in plants. These PAH-degraders can be valuable resource in constructing a PAH-degrading consortium. The objectives of this study were to isolate PAH-degrading endophytic bacteria from PAH-contaminated plants and to evaluate the capabilities of these bacteria for degrading PAHs in vitro, which will benefit the exploration of the re-colonization potential and PAH degradation performance of endophytic bacteria in target plants. The results will provide a new perspective in the reduction of plant PAH contamination risk in PAH-contaminated sites via the inoculation of plants with endophytic bacteria.

## 2. Materials and Methods

### 2.1. Isolation of PAH-Degrading Endophytic Bacteria

Healthy plants (*Conyza canadensis* and *Trifolium pretense* L.) were collected from PAH-contaminated sites near Sinopec Yangzi Petrochemical Co., Ltd. (Nanjing, China). Each plant sample was preserved at 4 °C until further use. Luria-Bertani (LB) medium containing 10.0 g/L of tryptone, 5.0 g/L of yeast extract, and 10.0 g/L of NaCl was used for the enrichment of PAH-degrading bacteria. Mineral salt (MS) medium was used as the basal medium for isolating PAH-degrading endophytic bacteria and evaluating the capabilities of these microbes for degrading PAHs. The MS medium contained 1.50 g/L of (NH_4_)_2_SO_4_, 1.91 g/L of K_2_HPO_4_·3H_2_O, 0.50 g/L of KH_2_PO_4_, 0.20 g/L of MgSO_4_·7H_2_O, and 1 mL of trace element solution (0.1 mg/L of CoCl_2_·6H_2_O, 0.425 mg/L of MnCl_2_·4H_2_O, 0.05 mg/L of ZnCl_2_, 0.01 mg/L of NiCl_2_·6H_2_O, 0.015 mg/L of CuSO_4_·5H_2_O, 0.01 mg/L of Na_2_MoO_4_·2H_2_O, and 0.01 mg/L of Na_2_SeO_4_·2H_2_O). PHE was utilized for the isolation of bacteria as a representative of PAHs. Plant tissues were sterilized after immersion in 75% (*v*/*v*) ethanol-water solution for 3–5 min and immersed in at 0.1% (*v*/*v*) mercuric chloride solution for 2–5 min. Subsequently, these plant tissues were washed with sterile deionized water at least three times to remove the surface sterilization agents and cultivated on an LB plate for confirmation that all external bacteria were eliminated [16,23]. After successfully surface disinfected, the plant tissues were aseptically ground.

The diluted solution was incubated in flasks containing 100 mL of MS media supplemented with 50 mg/L of PHE as previous study in our lab [24]. The aliquots were transferred weekly to fresh MS medium supplemented with increasing levels of PHE at least four times prior to the isolation of the bacterial strains (100 mg/L on the 2nd week, 150 mg/L on the 3rd week, and 200 mg/L on the 4th week) [14]. All flasks were incubated in the dark on a rotary shaker at 30 °C and 150 rpm. Isolation and purification procedures were performed on MS medium agar plates coated on the surface with a layer of 100 mg/L of PHE and subsequently incubated at 28 °C. The size and colour of the isolated colonies were recorded. The bacterial strains were selected based on colony morphology and colour.

Stock solutions of individual and PAH mixtures were prepared in methanol and used in all degradation experiments.

### 2.2. Identification of PAH-Degrading Endophytic Bacteria

The strains were classified based on 16S rRNA gene sequence analysis. The 16S rRNA gene fragments from the isolated strains were prepared according to the methods of Byers et al. [25]. Genomic DNA was used as a template to amplify the extracted 16S rRNA gene fragments through PCR, using the universal primers, 16S-27F and 16S-1492R (Invitrogen Co., Ltd., Shanghai, China). The amplification reactions were performed on a DNA Engine Thermal Cycler (PTC-200, BIO-RAD, Foster City, CA, USA). Sequencing was performed at the Nanjing Genscript Biotechnology Company, Ltd. (Nanjing, China). The 16S rRNA gene sequences were queried against the GenBank database [26], and the microgenetic analysis was performed using the Clustalx 1.83 and MEGA 6.0 programmes. Images of two strains were obtained using transmission electron microscope (H-7560, Hitachi, Japan). 

### 2.3. Biodegradation of PAHs Using Endophytic Bacteria

Strains P_1_ and P_3_ reflecting highly degrading-PAHs abilities were selected for further investigation. The cells were used as inocula in degradation studies after reaching the stationary phase through suspension in fresh MS medium at an optical density *OD*_600 nm_ of 1.0 (10^8^ CFU/mL). The degradation of PAHs was monitored in 50-mL flasks containing 20 mL of MS medium containing PAHs as the sole carbon sources, and 1-mL aliquots of the strain suspension were added to the prepared flasks. The control flasks were inoculated with sterilized MS medium to assess the abiotic effects on the stability of the PAHs. All cultures were incubated on a rotary shaker (150 rpm) at 28 °C for 7 days. Triplicate flasks from each treatment were retrieved for detection of the PAH concentrations.

#### 2.3.1. Degradation of PHE

To measure the PHE degradation of the strains in medium with different initial PHE concentrations, the 1-mL aliquots were suspended in 20 mL of MS medium supplemented with 50, 100, 150, 200, 300, 350, and 400 mg/L PHE as the sole carbon source, respectively.

#### 2.3.2. Capacities of Degrading Other PAHs

NAP, FLR, PYR, and B(*a*)P were selected as PAHs with 2-, 3-, 4-, and 5-ringed PAH representatives, respectively. The 1-mL aliquots were suspended in 20 mL of MS medium supplemented with NAP, FLR, and PYR (each at 100 mg/L), and 10 mg/L of B(*a*)P.

#### 2.3.3. Capacities of Degrading a Mixture of PAHs

The 1-mL aliquots were suspended in 20 mL of MS medium supplemented with a mixture of NAP, FLR, PHE, and PYR (each at 100 mg/L), and 10 mg/L B(*a*)P.

### 2.4. Effects of Additional Nutrients on Degradation of PHE

The 1-mL aliquots were suspended in 20 mL of MS medium supplemented with 100 mg/L PHE and each additional carbon or nitrogen nutrients. The levels of additional carbon nutrients were 10 g/L of glucose, fructose, sucrose, soluble starch, glycerine, mannite, and sorbitol; 5 g/L of yeast; 1 g/L of malic acid, oxalic acid, and citric acid; 150 mg/L of catechol and phthalate: and 300 mg/L of salicylic acid. The levels of additional nitrogen nutrients were 5 mg/L of NH_4_Cl, NH_4_NO_3_, (NH_4_)_2_SO_4_, (NH_4_)_2_HPO_4_, peptone, urea, and beef extract; and 1 mg/L of tryptophan, arginine, cysteine, and praline.

### 2.5. Detection of PAH Residues Using HPLC

The PAHs were extracted from the MS media with methyl alcohol, which was added to the medium at a ratio of 7:3 (*v*/*v*), ultrasonically extracted for 30 min, and centrifuged at 12,000× *g* for 10 min, followed by filtration through 0.22-µm filters [14]. The levels of PAHs in the prepared samples were quantified using an HPLC (Waters 600, Waters, Milford, MA, USA) equipped with a 4.6 × 150-mm reverse-phase C_18_ column using methanol/water (90:10) as the mobile phase at a flow rate of 0.8 mL/min. Chromatography was performed at 40 °C using a detection wavelength of 245 nm. For QA/QC, a standard sample was detected after every ten samples. 

### 2.6. Statistical Analyses

The statistical significance of any differences between treatments was subjected to one-way analysis of variance (ANOVA). Differences with *p* values < 0.05 were considered statistically significant. The kinetics equations for PAH degradation by strains P_1_ and P_3_ were calculated using regression analyses. Data analyses were performed using SPSS software (SPSS, Chicago, IL, USA).

## 3. Results and Discussion

### 3.1. Isolation and Identification of PAH-Degrading Endophytic Bacteria

**Figure 1:**
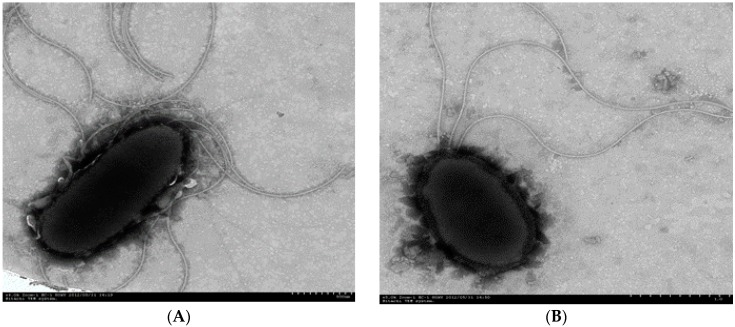
Electron micrographs of strain P_1_ ((**A**): ×6.0 K Zoom-1 HC-1 80 kV) and strain P_3_ ((**B**): ×5 K Zoom-1 HC-1 80 kV).

**Figure 2:**
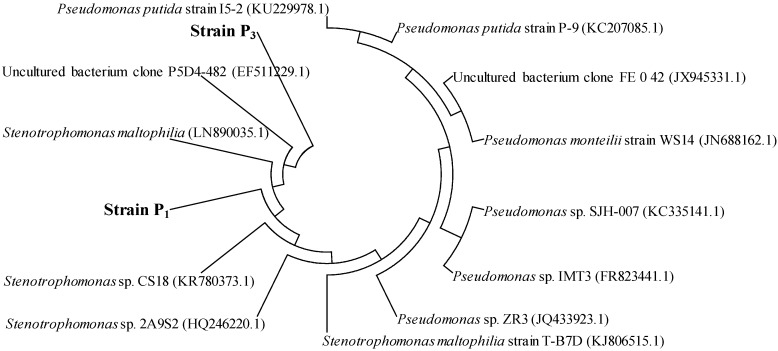
Phylogenetic analysis of isolated strains P_1_ and P_3_ based on 16S rRNA gene homology.

Strains P_1_ (GenBank KX594417) and P_3_ (GenBank KX594418) were isolated from *Conyza canadensis* and *Trifolium pretense* L., respectively, which could utilize PHE (up to 200 mg/L) as the sole source of carbon and energy. The cells of the two bacterial strains were short, nonsporing, acapsular, aerobic, and Gram-negative rods. Moreover, the cell size of strain P_1_ was ten times larger than that of strain P_3_ (Figure 1). Based on BLAST sequence comparison, the 16S rRNA of strain P_1_ was 99.98% similar to that of *Stenotrophomonas maltophilia*, while the 16S rRNA of strain P_3_ was 99.99% similar to that of *Pseudomonas monteilii* and 99.97% similar to that of *Pseudomonas plecoglossicida*. The phylogenetic tree shown in Figure 2 includes strains P_1_ and P_3_ and related species. These comparisons showed that strain P_1_ could be considered a *Stenotrophomonas* sp. strain, and strain P_3_ could be considered a *Pseudomonas* sp. strain. In previous reports *Pseudomonas* has been found in PAH- degrading consortia isolated from mangroves [15] and from leaf soil [27]. *Stenotrophomonas* has been previously reported to degrade high molecular weight PAHs [28]. 

### 3.2. Biodegradation Kinetics of PHE by Strains P_1_ and P_3_

**Figure 3:**
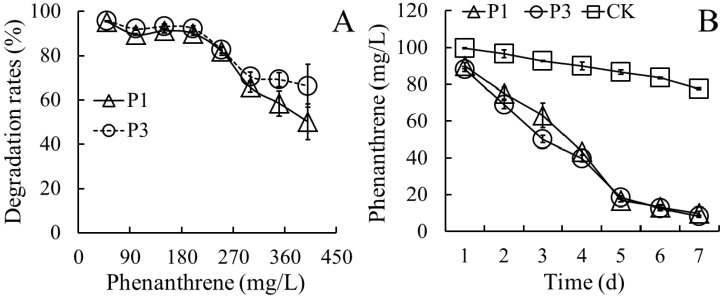
Degradation of different concentrations of PHE by the two bacterial strains (**A**) and the degradation of 100 mg/L PHE at different incubation times (**B**).

As shown in Figure 3A, PHE was effectively degraded by strains P_1_ and P_3_ after 7 days in MS medium supplemented with PHE at 50, 100, 150, 200, 250, 300, 350, and 400 mg/L, respectively. When the initial PHE levels in the medium were higher than 250 mg/L, more PHE was degraded by strain P_3_ than that degraded by strain P_1_. Strain P_3_ degraded PHE more efficiently than strain P_1_ when the levels of PHE in the media were higher than 250 mg/L. The degradation rates of PHE significantly decreased with increasing levels of PHE. When the levels of PHE in the media were less than 200 mg/L, the degradation of PHE by both strains was more than 90%. However, strain P_1_ degraded 81.8% PHE in medium containing 250 mg/L of PHE and 50.2% PHE in medium containing 400 mg/L of PHE. Strain P_3_ degraded 82.7% PHE in medium containing 250 of mg/L PHE and 66.4% PHE in medium containing 400 mg/L of PHE.

Additionally, we systematically evaluated the degradation kinetics of PHE (100 mg/L) by strains P_1_ and P_3_ after 7 days inoculation. PHE was rapidly degraded in the first 5 days (83.0% by strain P_1_ and 81.6% by strain P_3_), and subsequently the degradation rates decreased in the last 2 days (Figure 3B). 

The degradation kinetics equations were presented as

*C*_PHE_ = 167.61 × *e* ^−0.4075 t^ (by strain P_1_, r = 0.9738)
*C*_PHE_ = 156.21 × *e* ^−0.4099 t^ (by strain P_3_, r = 0.9871)

where *C*_PHE_ represents the residual concentrations of PHE in media (mg/L), and t represents the incubation time (days) [8].

PHE residues showed a significant negative correlation with bacterial counts, and the correlation coefficients were 0.985–0.973, suggesting that the efficiency of PHE degradation was positive correlated with the bacterial density. The half-life of PHE was 1.70 days for strain P_1_ and 1.69 days for strain P_3_, suggesting that the abilities of two strains to degrade PHE were very close.

### 3.3. Biodegradation Kinetics of Other PAHs

#### 3.3.1. Biodegradation of PAHs in Medium Containing Individual PAHs

**Figure 4:**
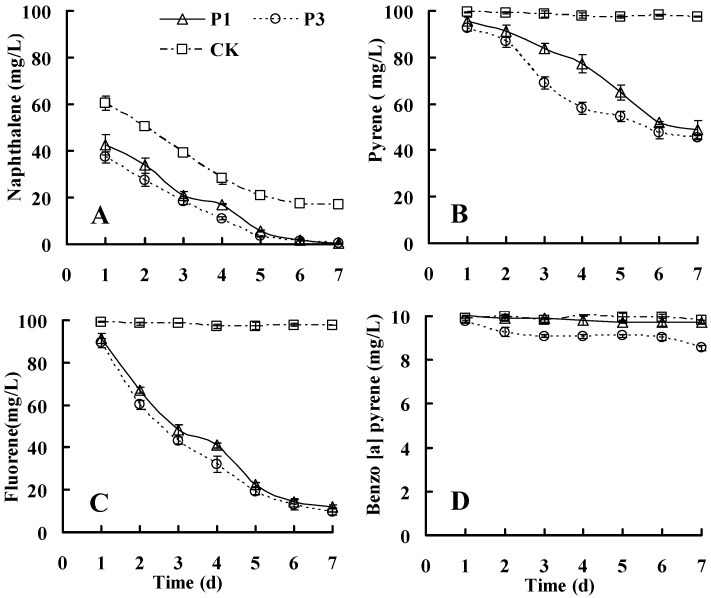
Degradation of NAP (**A**); PYR (**B**); FLR (**C**); and B(a)P (**D**). The strains were grown in MS medium containing single PAHs.

Both strains used NAP, FLR, PYR, and B(*a*)P as sole carbon resources. As shown in Figure 4, these strains had great capabilities of degrading NAP, FLR, and PYR, but lower capabilities of degrading B(*a*)P. Because strains P_1_ and P_3_ are aerobic bacteria, the biodegradation tests were performed under aerobic conditions. During the incubation period, a slight loss of PAHs occurred in the abiotic controls, excepting for NAP. As a two-ringed PAH, NAP was possibly removed from the MS medium through volatilization. After 24 h, the residual NAP in the medium decreased to 42.4 mg/L in the media for cultivating strain P_1_ and 37.27 mg/L in the media for cultivating strain P_3_, significantly less than the NAP levels in the control (60.5 mg/L). On day 7, the degradation rates were 98.6% by strain P_1_ and 98.1% by strain P_3_, except for the volatilizing part. In theory, the volatilization of NAP weakens with the decrease of NAP residual concentrations in MS medium. The NAP volatilization would be weaker in the media with versus without strains after 24 h cultivation. Our results suggested that both strains had considerable capabilities of degrading NAP within 24 h. This was also supported by the previous findings that NAP could be quickly degraded within 48 h [29]. The volatilization of PHE, FLR, PYR, and B(*a*)P was negligible (Figure 4). After 7 days, the residual levels of PHE, FLR, PYR, and B(*a*)P in the controls were 94.4, 97.5, 97.6, and 9.83 mg/L, respectively. The residual levels of PAHs in the media for cultivating strain P_1_ were 12.0 mg/L of FLR, 48.8 mg/L of PYR, and 9.74 mg/L of B(*a*)P, while those in the media for cultivating strain P_3_ were 9.5 mg/L of FLR, 45.4 mg/L of PYR, and 8.56 mg/L of B(*a*)P, suggesting that most PAHs were degraded by strains P_1_ and P_3_. Strain P_1_ degraded 98.6% NAP, 87.7% FLR, 50.0% PYR, and 0.9% B(*a*)P, while strain P_3_ degraded 98.1% NAP, 90.2% FLR, 53.5% PYR, and 12.9% B(*a*)P, which showed increased ability to degrade HMW-PAHs compared with *Pseudotrametes gibbosa* [21].

When each PAH was separately degraded by the two strains, the half-life values for NAP, PHE, FLR, PYR, and B(*a*)P, ranging from 0.86 to 135.88 days, increased with increasing number of benzene rings (Table 1), which is the same as other bacteria reported by Bacosa et al. [8]. 

Compared with strain P_1_, strain P_3_ reduced the half-life values for every PAH (PHE by 0.6%, FLR by 6.6%, PYR by 4.2%, and B(*a*)P by 58.9%), suggesting that strain P_3_ performed better than strain P_1_ in the degradation of PAHs according to the degradation fraction, which is an indicator of the metabolic capacities of PAH degradation as Yang et al. reported [30].

#### 3.3.2. Biodegradation of PAHs in Media Containing a Mixture of PAHs

**Figure 5:**
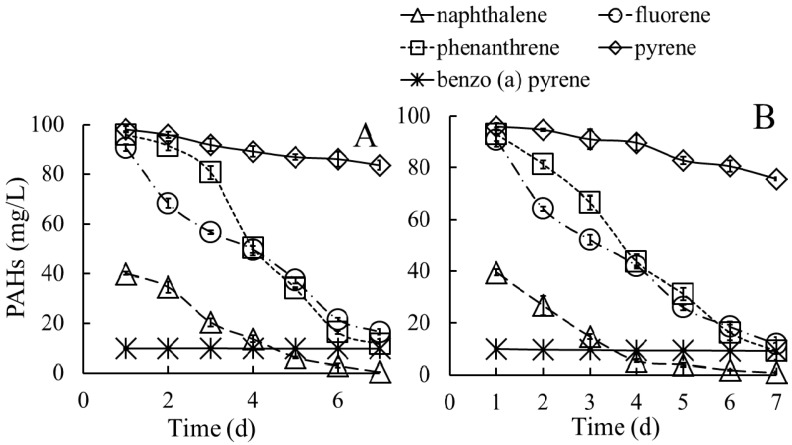
Degradation of PAHs by strains P_1_ (**A**) and P_3_ (**B**) in medium containing a mixture of PAHs.

After cultivation in a medium supplemented with a mixture of PAHs, both strains were able to simultaneously degrade the five representative PAHs. As reported by Bacosa and Inoue, pyrene was degraded faster as a sole substrate that in a mixture with fluorene and phenanthrene [8]. As shown in Figure 5, after cultivation in media containing a mixture of PAHs, strain P_1_ degraded 98.0% of NAP, 83.1% of FLR, 87.8% of PHE, 14.4% of PYR, and 1.6% of B(*a*)P, and strain P_3_ degraded 95.3% of NAP, 87.9% of FLR, 90.4% of PHE, 6.9% of PYR, and negligible B(*a*)P. The values of half-life for each PAH in media containing a mixture of PAHs ranged from 0.95 to 30 days (Table 2), suggesting that the PAHs were difficult to degrade in media containing a mixture of PAHs compared with media containing a single PAH, particularly for PYR and B(*a*)P. 

These results are consistent with the findings of Ma et al. [31], who suggested that the degradation of PYR and B(*a*)P could be competitively inhibited through other PAHs. Previous studies have reported that the presence of a mixture of PAHs could inhibit the production of 1-hydroxy-2-naphthoic acid, as a rate-limiting step in the degradation of PHE [32]. Compared with cultivation in media containing a single PAH, the half-life values generated through strain P_1_ increased 10.5% of NAP, 7.1% of PHE, 26.0% of FLR, 351.9% of PYR, and 142.9% of B(*a*)P. The half-life values generated through strain P_3_ increased 18.0% of NAP, 4.7% of PHE, 13.7% of FLR, 212.6% of PYR, 36.7% of B(*a*)P in media containing a mixture of PAHs. These results suggested that there were no co-metabolic effects among these PAHs.

### 3.4. Additional Carbon and Nitrogen Nutrients Enhance the Biodegradation of PHE

Additional carbon and nitrogen nutrients might enhance the degradation of PHE. As shown in Table 3, when glucose, fructose, sucrose, and yeast were supplemented in the media, the degradation rates of PHE by the two strains were more than 97% (*p* < 0.05). However, catechol, phthalate, and salicylic acid had no significant effects on the degradation of PHE by strain P_1_ and had little effect on PHE reduction by strain P_3_. The degradation rate of PHE by strain P_1_ was increased 8.1% through the addition of glycerine (*p* < 0.05). Furthermore, the degradation rates of PHE by strain P_3_ were increased 7.4%, 8.0%, and 7.6% upon the addition of soluble starch, mannite, and sorbitol in the medium, respectively (*p* < 0.05). These results suggested that multiple organic compounds would be beneficial for the degradation of recalcitrant HMW-PAHs by strains P_1_ and P_3_.

Several low-molecular-weight (LMW) co-metabolites enhanced the degradation of PAHs. LMW carbon sources, such as acetate and glucose, enhanced the deterioration of FLR, PHE, and PYR [33,34]; acetate, lactate, and mushroom compost markedly boosted the degradation of PHE [35]; yeast extract [36] and the residues and extracts of wood chips, bamboo leaves, and orange peels improved the degradation of HMW-PAHs [37]. According to the beneficial organic compounds provided in host plants, these two strains could degrade PAHs in internal plant tissues.

How did these additional nutrients improve the degradation of PAHs? Malic acid, oxalic acid, and citric acid enhanced the degradation of PHE through increasing the solubility of PHE, consistent with the results of Kobayashi et al. [38] and Chen et al. [39]. These authors reported that the degradation of PAHs was improved through some organic molecules, reflecting the increasing solubility of PAHs. Thus, there might be some alternative mechanisms for degrading PAHs when other organic carbon nutrients are added. Bhattacharya et al. [40] reported the development of an alternative novel biphasic process for the sustainable biodegradation of B(*a*)P under nutrient-sufficient culture conditions, with concomitant de novo ligninolytic enzyme expression.

In the present study, when additional beef extraction was added to the media, the maximum degradation rates of PHE was achieved at 99.1% (*p* < 0.05). Beef promoted biodegradation through providing nitrogen, carbon resources and growth factors for the bacterial strains. Moreover, the degradation of PHE could be significantly enhanced by other organic nitrogen nutrients, such as peptone and praline. However, inorganic nitrogen nutrients, such as NH_4_Cl, NH_4_NO_3_, (NH_4_)_2_SO_4_, and (NH_4_)_2_HPO_4_ had no significant effects on the degradation of PHE (*p* > 0.05). These results suggested that inorganic nitrogen nutrients could not promote the degradation of PAHs by the two strains when the nitrogen nutrients were sufficient, and these results were not consistent with the results of Vauramo et al. [41], who reported that nitrogen nutrients enhanced the degradation of PAHs. However, the biodegradation of PHE was reduced by tryptophan and arginine. As reported, tryptophan and arginine may inhibit the biofilm formation [42]. In this investigation, the respective growth of strains P_1_ and P_3_ was 43% and 40% less when tryptophan present, and the growth of strain P_1_ was 31% less when arginine present. 

### 3.5. Potential Application for PAH-Degrading Endophytic Bacteria

A novel technique, using PAH-degrading endophytic bacteria to remove PAHs in inner plants, has been documented in the last several years [14]. It has been proven that PAH-degrading endophytic bacteria, isolated from the plants grown in PAH-contaminated matrices, can easily re-colonize the host plant tissues and degrade the PAHs in the plants [14,43,44]. For instance, the endophytic bacterium *Pseudomonas* sp. Ph6-*gfp* could colonize the roots and shoot interiors of ryegrass after root inoculation; particularly, an increase in PHE biodegradation was observed in PHE-contaminated soil and in planta [14]. In the present study, both endophytic strains *Stenotrophomonas* sp. P_1_ and *Pseudomonas* sp. P_3_ were isolates from healthy plants (*Conyza canadensis* and *Trifolium pretense* L.) collected in PAH-contaminated sites, might have the potentials to re-colonize the target plants with PAH contamination. They effectively degrade test PAHs in vitro, indicating that these bacteria could be utilized to reduce the risk of plant PAH contamination in contaminated sites.

## 4. Conclusions

Endophytes with the capacity to highly degrade PAHs in vitro may have significant implications for recolonizing target plants at PAH-contaminated sites and reducing plant PAH residues. In our study, strains P_1_ (*Stenotrophomonas* sp.) and P_3_ (*Pseudomonas* sp.) were isolated from plants grown in contaminated soils, which could effectively degrade NAP, FLR, PHE, and PYR when exposed to PAHs individually or in a mixture. Moreover, additional organic carbon and organic nitrogen nutrients could significantly enhance the biodegradation of PAHs; therein, beef extract is an excellent co-metabolite. Compared with strain P_1_, strain P_3_ has more potential for application in the removal of PAHs from plant tissues. These results will provide a novel perspective for circumventing the risk of plant PAH contamination in PAH-contaminated sites through the inoculation of plants with endophytic bacteria.

## Figures and Tables

**Table 1:** Degradation kinetics equations for PAHs in media supplemented with individual PAHs.

PAHs	Degradation Analysis	Strain P_1_	Strain P_3_
NAP	Degradation kinetics equations	*C*_NAP_ = 189.09 × *e* ^−0.8072 t^ (r = 0.9353)	*C*_NAP_ = 138.99 × *e* ^−0.7813 t^ (r = 0.9681)
	Half-life (days)	0.86	0.89
PHE	Degradation kinetics equations	*C*_PHE_ = 167.61 × *e* ^−0.4075 t^ (r = 0.9738)	*C*_PHE_ = 156.21 × *e* ^−0.4099 t^ (r = 0.9871)
	Half-life (days)	1.70	1.69
FLR	Degradation kinetics equations	*C*_FLR_ = 137.99 × *e* ^−0.354 t^ (r = 0.9906)	*C*_FLR_ = 132.24 × *e* ^−0.3784 t^ (r = 0.9981)
	Half-life (days)	1.96	1.83
PYR	Degradation kinetics equations	*C*_PYR_ = 115.73 × *e* ^−0.1215 t^ (r = 0.9763)	*C*_PYR_ = 104.13 × *e* ^−0.127 t^ (r = 0.9829)
	Half-life (days)	5.70	5.46
B(*a*)P	Degradation kinetics equations	*C*_B(a)P_ = 10.036 × *e* ^−0.0051 t^ (r = 0.9356)	*C*_B(a)P_ = 9.7435 × *e* ^−0.0164 t^ (r = 0.8885)
	Half-life (days)	135.88	42.26

Where *C* represents the residual concentration of PAHs, mg/L; *t* represents the incubation time, days.

**Table 2:** Degradation kinetics equations for PAHs in media supplemented with a mixture of PAHs.

PAHs	Degradation Analysis	Strain P_1_	Strain P_3_
NAP	Degradation kinetics equations	*C*_NAP_ = 150.31 × *e* ^−0.7287 t^ (r = 0.9386)	*C*_NAP_ = 89.541 × *e* ^−0.6571 t^ (r = 0.9943)
	Half-life (days)	0.95	1.05
PHE	Degradation kinetics equations	*C*_PHE_ = 192.68 × *e* ^−0.3815 t^ (r = 0.9656)	*C*_PHE_ = 178.8 × *e* ^−0.3916 t^ (r = 0.9736)
	Half-life (days)	1.82	1.77
FLR	Degradation kinetics equations	*C*_FLR_ = 128.82 × *e* ^−0.2803 t^ (r = 0.9810)	*C*_FLR_ = 134.63 × *e* ^−0.3325 t^ (r = 0.9917)
	Half-life (days)	2.47	2.08
PYR	Degradation kinetics equations	*C*_PYR_ = 100.26 × *e* ^−0.0269 t^ (r = 0.9893)	*C*_PYR_ = 102.21 × *e* ^−0.0406 t^ (r = 0.9794)
	Half-life (days)	25.76	17.07
B(*a*)P	Degradation kinetics equations	*C*_B(*a*)P_ = 9.8059 × *e* ^‒0.0021 t^ (r = 0.6901)	*C*_B(*a*)P_ = 9.8931 × *e* ^−0.012 t^ (r = 0.9415)
	Half-life (days)	330	57.75

Where *C* represents the residual concentration of PAHs, mg/L, and *t* represents the incubation time, days.

**Table 3:** Effects of additional carbon and nitrogen nutrients on the degradation of PHE.

Extra Carbon Sources			Extra Nitrogen Sources		
Carbon Sources	Degradation (%)		Nitrogen Sources	Degradation (%)	
	Strain P_1_	Strain P_3_		Strain P_1_	Strain P_3_
CK	90.5 ± 0.2 e	90.4 ± 0.5 h	CK	90.5 ± 0.2 c	90.4 ± 0.5 cd
Glucose	98.7 ± 0.1 a	98.7 ± 0.1 ab	NH_4_Cl	90.0 ± 1.2 c	91.6 ± 1.5 cd
Fructose	99.0 ± 0.1 a	98.8 ± 0.2 ab	NH_4_NO_3_	89.7 ± 1.4 c	91.1 ± 0.7 cd
Sucrose	97.7 ± 0.2 a	97.9 ± 0.4 b	(NH_4_)_2_SO_4_	91.4 ± 0.8 c	90.1 ± 1.1 d
Yeast	98.4 ± 0.1 a	99.3 ± 0.1 a	(NH_4_)_2_HPO_4_	91.2 ± 1.6 c	90.9 ± 1.4 cd
Soluble starch	94.1 ± 0.5 c	97.8 ± 0.2 b	Peptone	96.7 ± 0.9 b	99.0 ± 0.2 a
Glycerine	98.6 ± 0.1 a	94.8 ± 0.3 c	Urea	90.1 ± 1.1 c	89.6 ± 0.9 d
Malic acid	95.8 ± 0.5 b	92.8 ± 0.7 de	Beef extract	99.1 ± 0.2 a	99.1 ± 0.1 a
Oxalic acid	95.2 ± 0.5 b	93.0 ± 0.3 de	Tryptophan	76.1 ± 2.6 e	66.2 ± 2.5 e
Citric acid	96.1 ± 0.5 b	93.3 ± 0.6 d	Arginine	84.6 ± 1.3 d	90.1 ± 2.5 d
Mannite	92.7 ± 0.6 d	98.4 ± 0.3 ab	Cysteine	91.4 ± 1.0 c	92.5 ± 1.6 c
Sorbitol	92.5 ± 0.5 d	98.0 ± 0.2 b	Proline	94.7 ± 1.1 b	95.7 ± 0.5 b
Catechol	91.2 ± 0.5 de	92.1 ± 0.4 ef			
Phthalate	90.9 ± 0.5 e	90.8 ± 1.0 gh			
Salicylic acid	90.5 ± 1.0 e	91.6 ± 0.7 fg			

Same lowercase letters indicate lack of statistically significant differences within the same line (*p* < 0.05).
